# Sternal sparing aortic valve replacement via right anterior minithoracotomy: An early experience

**DOI:** 10.1007/s12055-023-01596-3

**Published:** 2023-10-03

**Authors:** Rong Hui (Misté) Chia, Pragnesh Joshi

**Affiliations:** 1 Department of Cardiothoracic Surgery, Sir Charles Gairdner Hospital, Hospital Ave, Nedlands, WA 6009 Australia; 2 St John of God Subiaco Hospital, Subiaco, WA Australia; 3 Hollywood Private Hospital, Nedlands, WA Australia; 4 Mount Hospital, Perth, WA Australia; 5 University of Western Australia, Perth, WA Australia

**Keywords:** Aortic valve replacement, Minimally invasive, Right anterior minithoracotomy, Sternal sparing

## Abstract

**Purpose:**

This study aims to evaluate the perioperative outcomes of aortic valve replacement (AVR) via right anterior minithoracotomy (RAT) during the learning curve.

**Methods:**

It was a retrospective, observational, cohort study of patients who underwent RAT AVR from June 2015 to April 2022. Primary outcomes measured were 30-day morbidity and mortality.

**Results:**

A total of 107 consecutive patients underwent elective RAT AVR. Our patients were mostly male (78.5%), elderly (mean 68.7 years), and obese (34.6%). A majority of the patients (93.5%) were of low operative risk. Median cross-clamp and bypass times were 95 and 123 minutes respectively. There was a statistically significant correlation between increase in number of cases and decrease in operative time. All patients had no paravalvular leak at discharge. There were no operative cardiovascular mortality or major morbidity including stroke, myocardial infarction, renal failure requiring dialysis, or vascular complication. No patient required intraoperative conversion to full sternotomy for completion of AVR.

**Conclusion:**

Our study demonstrated that RAT AVR can be safely introduced. The learning curve required in performing RAT AVR can be safely negotiated through training, previous experience in minimally invasive surgery, careful patient selection including use of preoperative computed tomography of the aorta, and introduction of sutureless/rapid deployment valves.

## Introduction

Sternal-sparing aortic valve replacement (AVR) has evolved in the last few decades and the conduct of AVR is now possible through a limited (5–7 cm) right-sided incision. However, AVR through right anterior minithoracotomy (RAT) is still not widespread due to the associated technical challenges and the required steep learning curve. This is the largest Australian study to date, evaluating the outcomes of RAT AVR during the learning curve.

## Materials and methods

### Patient selection and data collection

This was a retrospective, observational, cohort study of prospectively collected data from 107 consecutive patients who underwent RAT AVR at four institutions by a single surgeon from June 2015 to April 2022. Data was collected from patient records, anesthetic and perfusion charts. Outcomes measured were operative morbidity and mortality within 30 days of the operation.

### Preoperative planning and surgical technique

Patients with significant atheroma in the aortic arch or descending thoracic aorta based on preoperative computed tomography (CT) were excluded due to the risk of retrograde embolization in the initial experience, when femoral artery was routinely used for inflow cannulation. Subsequently when direct aortic cannulation was routinely performed, only those with substantial ascending aortic atheroma were excluded from the RAT procedure. Patients were also considered unsuitable if they had substantial pectus, or if they required concomitant coronary arterial bypass grafting or significant aortic procedure.

**Fig. 1:**
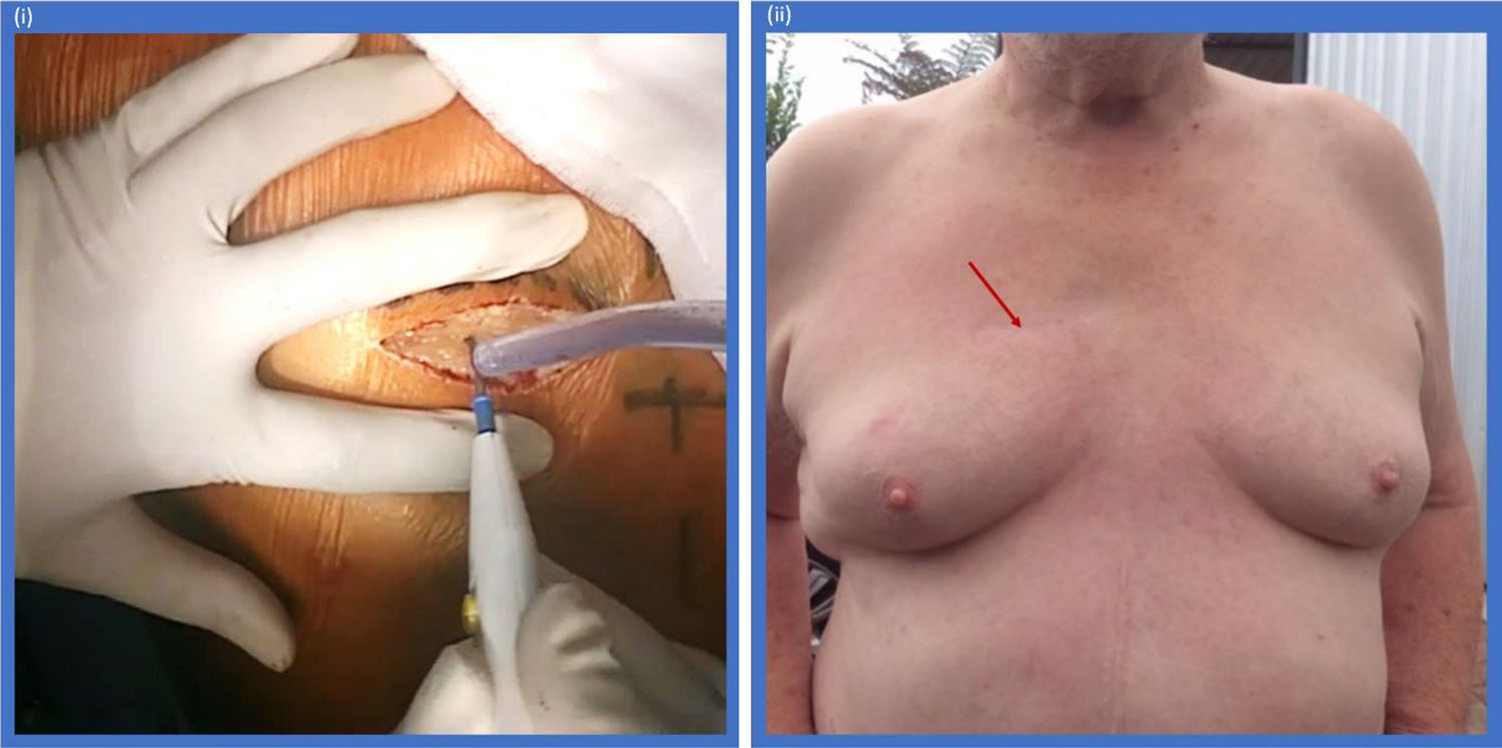
(i) A 5-cm incision for RAT AVR intraoperatively. (ii) Incision wound postoperatively, demonstrating outstanding cosmesis

**Fig. 2:**
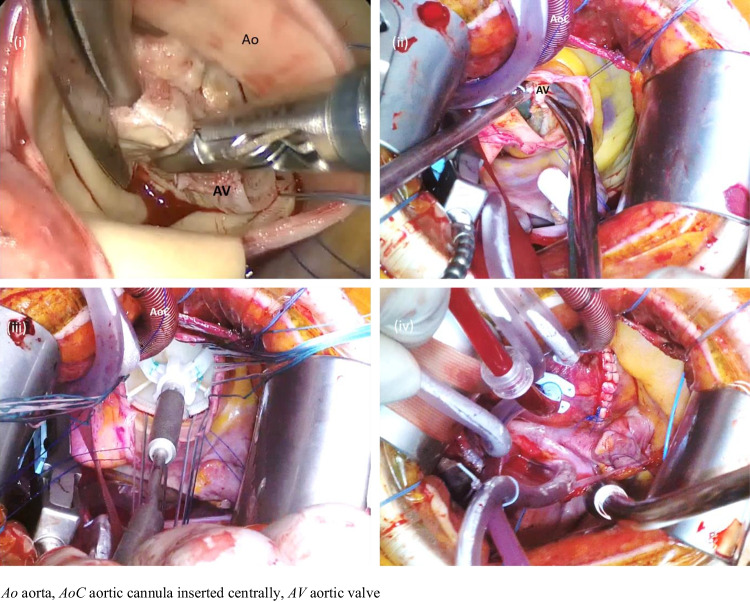
Intraoperative views through a RAT incision. The focussed view via RAT incision still provided good exposure of pertinent mediastinal structures including the AV (i and ii) and allowed surgical removal of the pathological valve with subsequent thorough surgical debridement of any calcification present; and a full view of the closed aortotomy incision (iv) to check for hemostasis, demonstrating the safety of RAT AVR. Even when aortic cannulation was established centrally (ii and iii), it did not obstruct operative view

RAT technique was performed with a 5-cm incision in the second intercostal space from the lateral border of the sternum (Fig. 1). The third sternocostal cartilage was divided, and the right internal mammary artery and vein were ligated. Aortic cannulation was achieved peripherally through the transfemoral approach. With increasing experience, central aortic cannulation was performed primarily for subsequent cases, while still maintaining excellent operative field (Fig. 2). Venous cannulation was established percutaneously with Seldinger technique under transesophageal echocardiographic (TOE) guidance through the right common femoral vein using a multi-staged venous cannula.

### Statistical analysis

Statistical analysis was performed using IBM® SPSS® Statistics (Herrenberg, Germany). Continuous variables were expressed as mean ± standard deviation for normally distributed data and median (interquartile ranges) for non-normal data. Categoric data was presented with actual values and percentage of the total. Analysis of the surgeon’s level of experience was completed as a categorical variable with patients chronologically assigned to groups of tens. Differences in operative times were compared using 1 factorial analysis of variance. Statistical significance was defined as a *p* value of less than 0.05.

## Results

From June 2015 to April 2022, a total of 107 patients underwent RAT AVR.

### Baseline characteristics

The clinical characteristics of the study population are detailed in Table 1. Our patient population was relatively elderly (mean age 68.7 ± 9.6 years; 65–80 years 65.4%; and > 80 years 7.5%). About a third of our patients (34.6%) had a body mass index (BMI) of more than 30 kg/m^2^. Most of our cohort (93.5%) were of low operative risk.

**Table 1:** Baseline characteristics

	RAT (*n* = 107)	
Age		68.7 ± 9.6
Male		78.5% (84)
BMI (kg/m^2^)		28.3 ± 4.4
Underweight	0% (0)	
Normal	23.4% (25)	
Overweight	42.0% (45)	
Obese	34.6% (37)	
Smoker		51.4% (55)
Current	10.3% (11)	
Respiratory disease		15.0% (16)
Diabetes		15.9% (17)
Insulin-dependent	3.7% (4)	
Hypercholesterolemia		70.1% (75)
Hypertension		65.4% (70)
Arrhythmia		12.1% (13)
Pacemaker in-situ		3.7% (4)
Previous Cardiothoracic intervention		0.9% (1) *1 repair of aortic coarctation*
Previous Cardiology intervention		11.2% (12) *11 PTCA/stent;* 1 balloon valvuloplasty of AV
Cerebrovascular disease		2.8% (3) *3 stroke (remote)*
Peripheral arterial disease		7.5% (8)
Renal dysfunction requiring dialysis		0% (0)
IE		4.7% (5) *4 treated;* *1 active*
Immunosuppressed		2.8% (3)
Previous MI		5.6% (6)
Presence of significant CAD on preoperative imaging		0% (0)
NYHA functional class		1.8 ± 0.7
I/II	89.7% (96)	
III/IV	10.3% (11)	
LVEF		
Normal		75.7% (81)
Mildly impaired		13.1% (14)
Moderately impaired		11.2% (12)
Severely impaired		0% (0)
Medication		
Anticoagulation		2.8% (3)
Antiplatelet		43.0% (46)
Steroid		0.9% (1)
STS-PROM/%		1.4 (1.0, 2.3)
Low (< 4%)	93.5% (100)	
Intermediate (≥ 4%)	5.6% (6)	
High risk of mortality (≥ 8%)	0.9% (1)	
Urgency		
Elective		100% (107)
Urgent		0% (0)
Emergency		0% (0)
Salvage		0% (0)
Primary AV pathology		
AS		68.2% (73)
AR		15.0% (16)
Mixed		16.8% (18)

*AR* aortic regurgitation, *AS* aortic stenosis, *AV* aortic valve, *BMI* body mass index; *CAD* coronary arterial disease, *CCS* Canadian Cardiovascular Society Angina Grading Scale, *IE* infective endocarditis, *LVEF* left ventricular ejection fraction, *MI* myocardial infarction, *NYHA* New York Heart Association, *STS-PROM* Society of Thoracic Surgeons predicted risk of 30-day mortality, *TIA* transient ischaemic attack

### Operative data

Surgical details are summarized in Table 2. Arterial cannulation was established peripherally through the common femoral artery for 27 patients (25.2%) and centrally through the ascending aorta for 80 patients (74.8%). Patients were observed to have either tricuspid (49.5%) or bicuspid (47.7%) valve. Unicuspid valve was uncommon (2.8%). Most patients received tissue valve implantation (87.9%).

**Table 2:** Operative variables

	RAT (*n* = 107)
Arterial cannulation site	
Central	74.8% (80)
Peripheral (CFA)	25.2% (27)
Venous cannulation site	
Central	0% (0)
Peripheral	100% (107)
AV type	
Tricuspid	49.5% (53)
Bicuspid	47.7% (51)
Unicuspid	2.8% (3)
Type of prosthesis	
Tissue	87.9% (94) *of which 66.0% (62) sutured AVR, 11.7% (11) sutureless AVR, and 22.3% (21) rapid deployment AVR*
Mechanical	12.1% (13)
Prosthesis size/mm	24.1 ± 2.2
Concomitant procedures	11.2% (12) *6 ascending aortoplasty;* *1 VATS left lower lobe wedge resection;* *1 right middle lobe biopsy and thymic lymph node biopsy;* *3 VAT LAAO; and* *1 thoracoscopic left atrial ablation* + *bilateral pulmonary vein isolation* + *LAAO*
Cross-clamp time/min	95 (80, 119)
Need for second cross-clamp	0.9% (1) *for paravalvular leak requiring redo-AVR*
CPB time/min	123 (109, 145)

*AV *aortic valve, *AVR *aortic valve replacement, *CFA* common femoral artery, *CPB* cardiopulmonary bypass, *LAAO* left atrial appendage occlusion, *VAT* video-assisted thoracoscopic

Twelve patients (11.2%) had concomitant procedures at the time of RAT AVR as detailed in Table 2. Eleven patients (10.3%) had significant calcification requiring extensive decalcification of aortic root, left ventricular outflow tract, interventricular septum, aortomitral curtain, and/or anterior mitral leaflet, with 1 patient requiring reconstruction of aortomitral curtain and base of anterior mitral leaflet using pericardial patch. One patient had an aortic root enlargement.

**Fig. 3:**
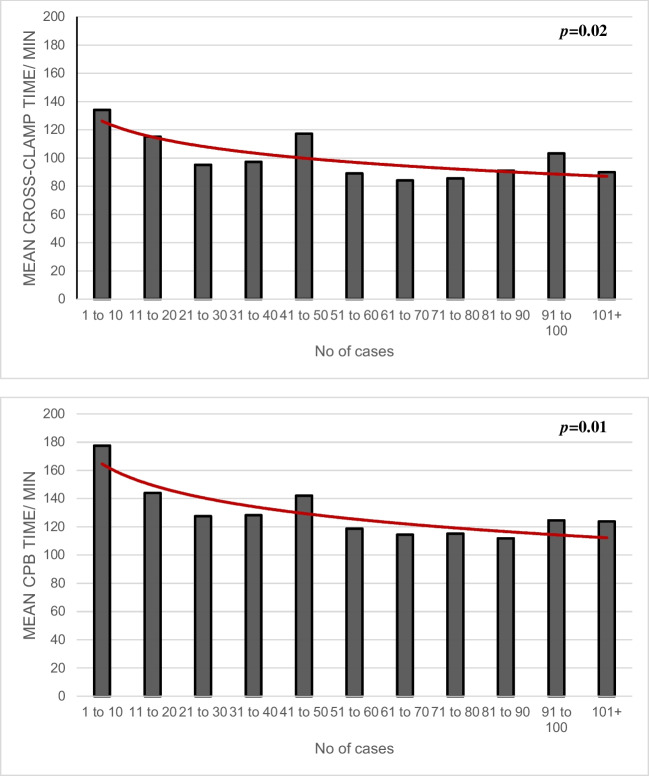
Operative times according to surgeon’s experience

Operative time according to surgeon’s experience is illustrated in Fig. 3. As demonstrated, there was a statistically significant decline in both cross-clamp time (134 minutes in initial period vs 90 minutes in latest period, *p* = 0.02) and cardiopulmonary bypass time (178 minutes in initial period vs 124 minutes in latest period, *p* = 0.01) with increasing operative experience. Overall, median cross-clamp and cardiopulmonary bypass time were 95 and 123 minutes respectively. One patient (0.9%) required second cross-clamping to repair mild paravalvular leak. All patients had good aortic valvular function with no paravalvular leak confirmed on intraoperative TOE after weaning off bypass and on predischarge echocardiogram.

### Clinical outcomes

**Fig. 4:**
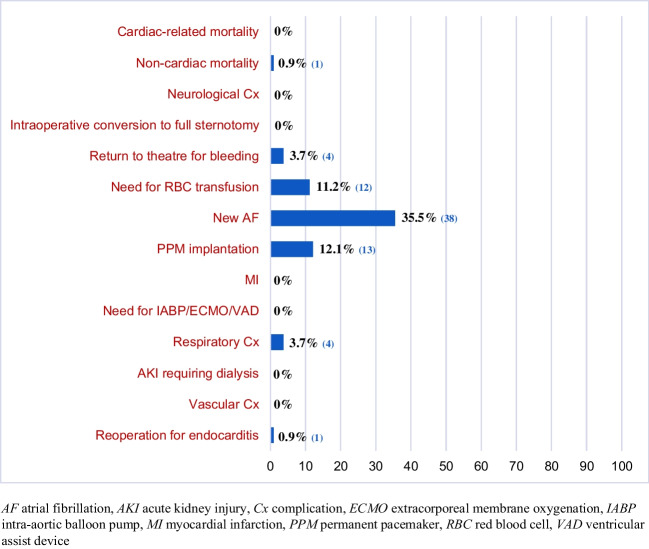
Postoperative (major and minor) morbidities and mortality

Clinical outcomes at 30 days are shown in Fig. 4. Perioperatively, there were no cases of cardiovascular mortality or stroke. There was 1 non-cardiac death. This patient died from respiratory failure after being readmitted for acute exacerbation of pre-existing interstitial lung disease.

None of the patients required conversion to full sternotomy intraoperatively for completion of RAT AVR. Four patients (3.7%) required reoperation for postoperative bleeding, of which 2 cases had sternotomy while the other 2 patients had reoperation via the same thoracotomy incision in later part of our series. Twelve patients (11.2%) required red blood cell transfusion. Drain output at 4 hours was 130 (75–200) ml. At the time of discharge, mean hemoglobin level was 111.7 ± 16.8 g/L.

Atrial fibrillation occurred in 38 (35.5%) patients. Thirteen (12.1%) patients required permanent pacemaker (PPM) implantation, of which 6 patients had balloon expandable sutureless or rapid deployment valves. For patients who required PPM implantation, 2 patients had complete heart block while 11 patients had tachy-brady syndrome and received PPM for safe administration of rate-controlling medications. At post-discharge follow up, device check was again performed on these patients which confirmed that only 2 patients had persistent complete heart block requiring PPM.

Three patients (2.8%) required prolonged ventilation and 1 (0.9%) patient required reintubation for respiratory failure due to obesity and pre-existing obstructive sleep apnea. One patient (0.9%) had infective endocarditis requiring reoperation 24 days after the initial surgery. As shown in Fig. 4, there were no cases of myocardial infarction, need for mechanical circulatory support, acute renal injury requiring dialysis, or vascular complication.

Mean duration of intensive care unit (ICU) stay was 2 days (2.4 ± 1.7 days). Of note, more than half of our patients were operated in private facilities where patients were routinely observed in ICU for at least 48 hours. Mean duration of hospital stay was 8 days (7.6 ± 3.4 days).

## Discussion

Despite its advantages, RAT AVR is still not widely adopted. This may largely be attributed to the required additional training and the associated steep learning curve. Our study, which is the largest Australian report of its kind, has demonstrated that RAT AVR may be safely introduced as a routine procedure while getting over the learning curve.

While rates of mortality and stroke for full sternotomy AVRs have previously been reported to be between 1.4–1.9% and 1.2–1.3% respectively on national databases [1, 2], there were no cases of cardiovascular mortality or stroke in our patients. The zero incidence of perioperative stroke observed in our study was likely the result of careful patient selection (which involved the use of preoperative gated CT aortogram) and intraoperative conduct of central aortic cannulation in majority of our patients. Hence, we suggest the routine use of CT aortogram for planning of RAT AVR to minimize the occurrence of perioperative stroke.

The avoidance of sternotomy in RAT AVR has eliminated sternal-related complications, reduced pain and decreased loss of blood while providing better cosmesis, and increased patient satisfaction. Although deep sternal wound infections are uncommon in isolated full-sternotomy AVR, with a reported incidence of only 0.7% in Australasia [1], they are still associated with poorer outcomes including increased mortality [3]. With the sternal-sparing approach in RAT AVR, the risk of sternal infection was fully eradicated with no cases of postoperative wound infection observed in our patients, which is particularly beneficial in patients with elevated BMI. Despite having a large proportion of patients with a BMI of more than 25 kg/m^2^, none of our patients required intraoperative conversion to full sternotomy for completion of AVR. In addition, it is possible to perform RAT AVR without division of the third sternocostal junction but this may limit exposure and necessitate femoral arterial cannulation, adding to the risk of perioperative stroke. In our experience, the division of costal cartilages did not result in any major adversity or significant pain issues. There were no formal measurements of postoperative pain or mobility in our study. However, we observed that our patients experienced lesser pain as compared to other minithoracotomy approaches in our unit. Also, we notice a reduced requirement for red blood cell transfusion in our patients, which was half of the reported binational average for isolated AVR (11.2% vs 25.8%) [1]. This may be due to the complete preservation of sternum in RAT AVR. Mean hemoglobin level at time of discharge was more than 100 g/L, indicating minimal blood loss during or post-RAT surgery.

Operative time was longer in our series as observed in other minimally invasive surgeries. Nevertheless, it is important to note that this has not translated into adverse clinical outcomes. In addition, operative time in our study has reduced considerably while getting over the learning curve. This was mainly attributed to training and cumulative experience with performing RAT AVR over the years. Other factors which helped overcome the learning curve safely included previous experience in other minimally invasive surgeries, careful patient selection and preoperative planning involving the use of CT aortogram, as well as the introduction of rapid deployment and sutureless valve technology to decrease operation time.

With continued advancements in medical technology, transcatheter aortic valve implantation (TAVI) has emerged as a potential alternative to surgical AVR [4] and its application has now been extended to the low surgical risk group [5, 6]. The emergence of TAVI has led to an increased interest in sternal-sparing approaches but there are no large randomized trials directly comparing the outcomes of RAT AVR with TAVI. Some ongoing concerns with TAVI use include its risk of paravalvular leak, its limited application in bicuspid aortic stenosis, and the lack of data to demonstrate its long-term durability [5, 6]. We believe that a well-established RAT AVR program is an ideal alternative to percutaneous valves, especially for young patients with low operative risks or patients who are unsuitable candidates for TAVI.

While performing a successful RAT AVR is demonstrated to be achievable in our study, having it widely adopted remains challenging due to institutional and surgeon-related factors. Institutions need to be willing to invest in training and in purchasing new equipment, while surgeons have to maintain an open mind to perform minimally invasive AVR despite the already well-performed low-risk full sternotomy AVR. It is possible to develop technical skills to get over the learning curve of RAT AVR without compromising patient outcomes.

## Limitations of study

We acknowledge the retrospective nature of this study, which has its limitations adherent to its study design. Also, this study has evaluated only a single surgeon’s technique and outcomes. Therefore, the outcomes here may not necessarily apply for every other surgeon. However, the technique used is well-defined and so should be reproducible.

## Conclusions

In summary, our study has demonstrated that RAT AVR may be safely introduced as a routine procedure after getting over the learning curve. It is possible to overcome the learning curve safely with training, careful patient selection and preoperative planning, cumulative operative experience with RAT AVR, and use of rapid deployment or sutureless aortic valves. Although performing RAT AVR involves relatively long operative time and requires a steep learning curve, it provides good cosmesis, preserving sternal integrity with no significant impact on major clinical outcomes.
